# Isolation, culture, and characterization of chicken intestinal epithelial cells

**DOI:** 10.1186/s12860-021-00349-7

**Published:** 2021-02-12

**Authors:** Federico Ghiselli, Barbara Rossi, Martina Felici, Maria Parigi, Giovanni Tosi, Laura Fiorentini, Paola Massi, Andrea Piva, Ester Grilli

**Affiliations:** 1 DIMEVET, University of Bologna, Via Tolara di Sopra, 50, Ozzano dell’Emilia, 40064 Bologna, BO Italy; 2 Vetagro S.p.A., Via Ignazio Porro, 2, 42124 Reggio Emilia, RE Italy; 3 Istituto Zooprofilattico Sperimentale Della Lombardia e Dell’Emilia Romagna, Sede Territoriale di Forlì, Via Don Eugenio Servadei, 47122 Forlì, FC Italy; 4 Vetagro, Inc., 116 W. Jackson Blwd., Suite #320, Chicago, IL 60604 USA

**Keywords:** Chicken, Enterocyte, Immunofluorescence, Embryo, Primary culture

## Abstract

**Background:**

Enterocytes exert an absorptive and protective function in the intestine, and they encounter many different challenging factors such as feed, bacteria, and parasites. An intestinal epithelial in vitro model can help to understand how enterocytes are affected by these factors and contribute to the development of strategies against pathogens.

**Results:**

The present study describes a novel method to culture and maintain primary chicken enterocytes and their characterization by immunofluorescence and biomolecular approaches. Starting from 19-day-old chicken embryos it was possible to isolate viable intestinal cell aggregates that can expand and produce a self-maintaining intestinal epithelial cell population that survives until 12 days in culture. These cells resulted positive in immunofluorescence to Cytokeratin 18, Zonula occludens 1, Villin, and Occludin that are common intestinal epithelial markers, and negative to Vimentin that is expressed by endothelial cells. Cells were cultured also on Transwell® permeable supports and trans-epithelial electrical resistance, was measured. This value gradually increased reaching 64 Ω*cm^2^ 7 days after seeding and it remained stable until day 12.

**Conclusions:**

Based on these results it was confirmed that it is possible to isolate and maintain chicken intestinal epithelial cells in culture and that they can be suitable as in vitro intestinal model for further studies.

**Supplementary Information:**

The online version contains supplementary material available at 10.1186/s12860-021-00349-7.

## Background

The chicken intestinal epithelium is made of a single-cell monolayer, the most important barrier against the outer environment [1]. At the same time, these cells are also essential for the proper absorption of substances deriving from feed digestion [2]. In literature, most of the in vitro studies use immortalized mammalian cell lines (such as the human intestinal cell line Caco-2) which are cheap and easy to manage and maintain, but these cells may not reproduce the full profile of an in vivo differentiated epithelium [3]. Moreover, mammalian cells are less suitable for the study of chicken intestinal physiology or the interaction of specific intestinal pathogens with enterocytes, for example, coccidia or *C. perfringens* [4]. For these reasons, an avian intestinal epithelial cell model can prove itself as a useful tool, but only a few isolation protocols for chicken intestinal cells have been developed so far and  none succeeded at maintain a fully characterized monolayer in culture or more than 7–10 days [5–13]. This study aimed to establish a novel reproducible protocol to obtain and characterize a primary chicken intestinal epithelial cells culture (cIECs) that can last longer than the normal enterocyte turnover of about 3–5 days.

## Results

### cIEC isolation and culture

**Fig. 1:**
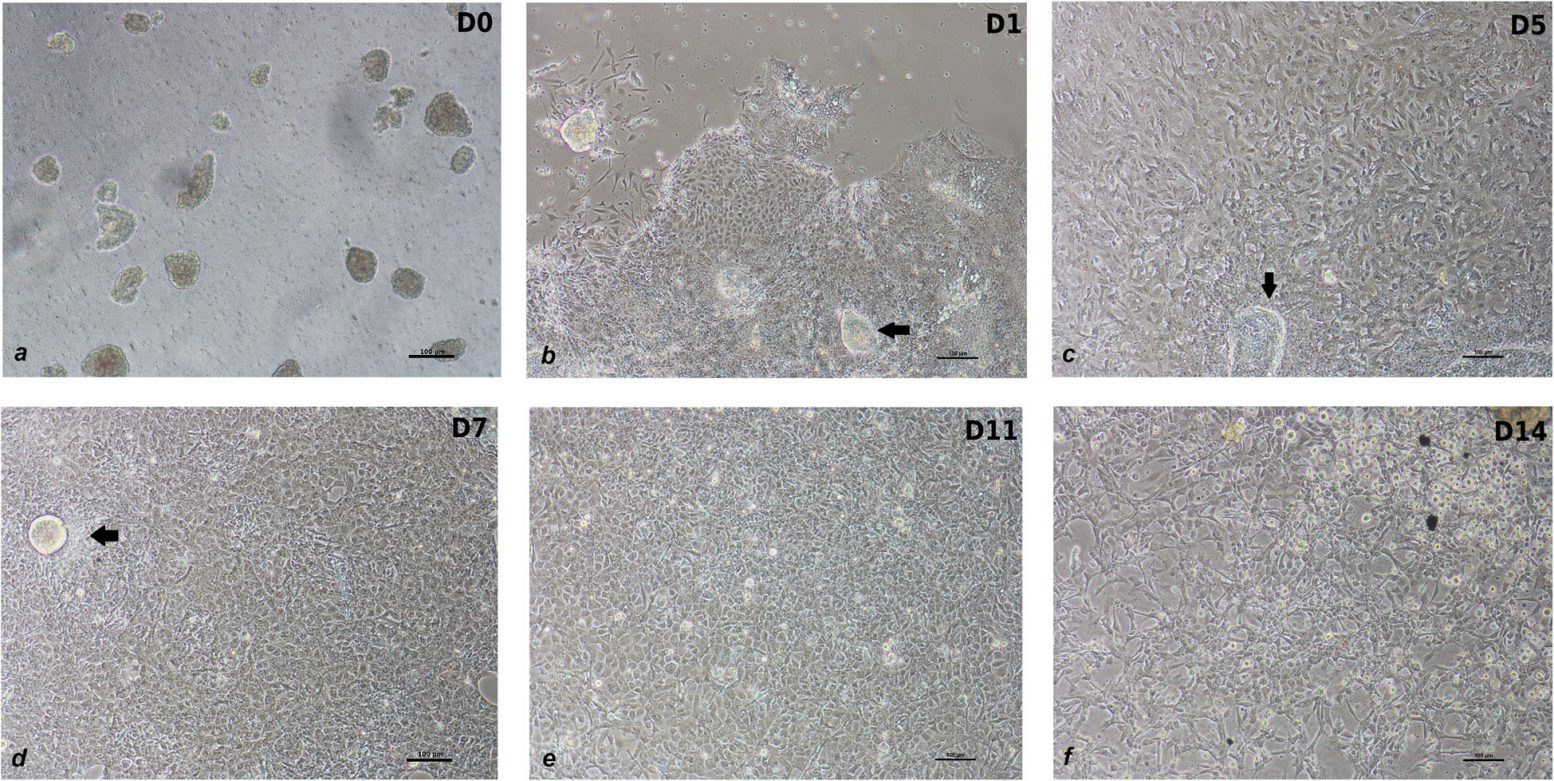
cIECs culture at different time points. **a** spheroids during seeding; **b** 24 h after seeding; **c** 5 days after seeding; **d** 7 days after seeding; **e** 11 days after seeding; **f** 14 days after seeding. Black arrows in sections b, c, d indicate foci of proliferation

Intestinal cell aggregates rapidly attached to the Matrigel® coated-wells (Fig. 1a) and cells gradually migrated out from the foci of proliferation within 24 h in culture, as it is visible in Fig. 1b. Cells continued to divide until 5 days after seeding when they reached confluency (Fig. 1c). After 7 days in culture, cells appeared with a cobblestone morphology (Fig. 1d) and remained confluent until day 11 (Fig. 1e). Then, the epithelium started to degenerate, and spaces among cells were visible on day 14 (Fig. 1f).

### Contaminating cell removal

**Fig. 2:**
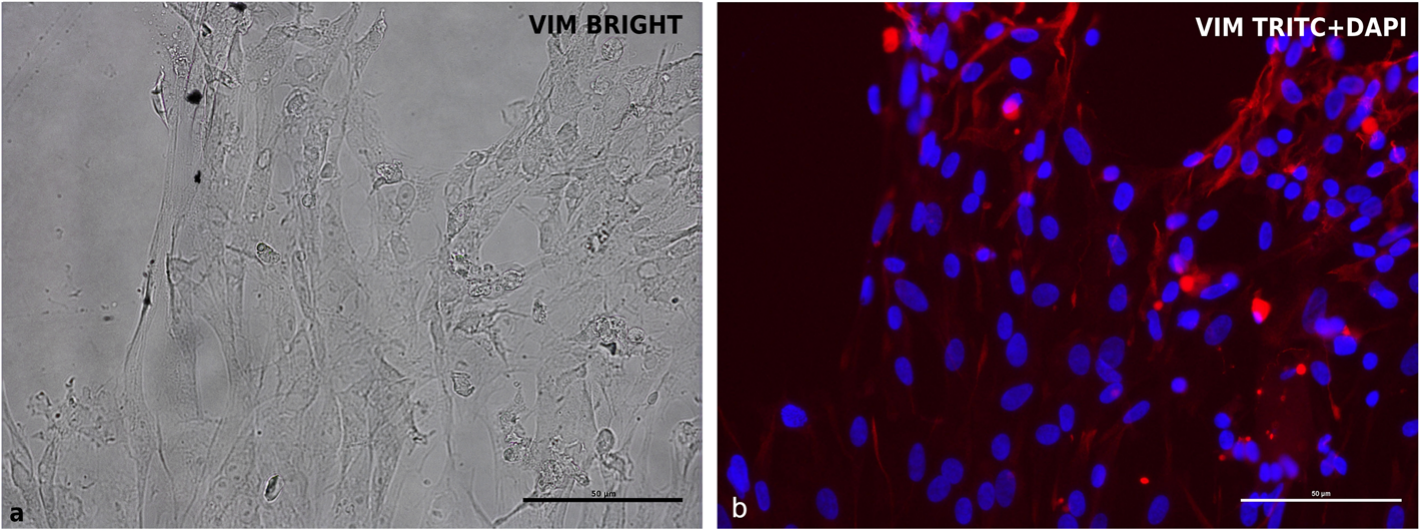
VIM expression by IF in chicken embryonic endothelial cells. **a** brightfield; **b** VIM (TRITC+DAPI). This analysis was performed to test the efficacy of VIM antibody against chicken cells

**Fig. 3:**
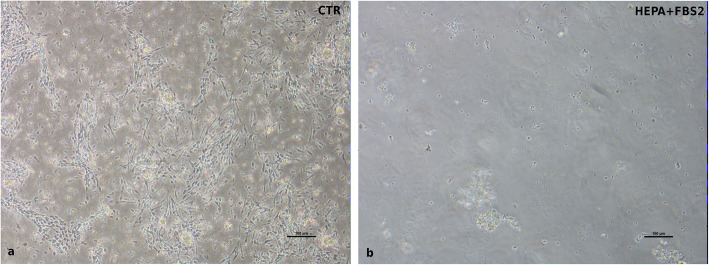
Contaminating cells cultured on Matrigel® matrix (0.8 mg/mL) coated wells with and without heparin and reduced FBS (2%). **a** CTR = FBS 10% only (24 h after seeding); **b** HEPA+FBS2 = FBS 2% and 0.1 mg/mL heparin sodium salt (24 h after seeding)

Typical contaminating cells (endothelial cells, myofibroblasts, and fibroblasts) were obtained recovering the < 40 μm fraction from reverse filtration, as explained in the cIECs isolation procedure paragraph. These cells resulted positive for Vimentin (VIM) in immunofluorescence (IF), as showed in Fig. 2, (Fig. 2) and different endothelial and fibroblastic cell markers in PCR (see Additional file 1). In Fig. 3 (Fig. 3) it is reported the comparison between isolated cells cultured with or without heparin sodium salt + 2%FBS (HEPA+FBS2). It is evident how HEPA+FBS2 was able to prevent the adhesion of various contaminating cells on Matrigel® matrix coated wells (Fig. 3b). Cells cultured without heparin and with 10% FBS grew normally (Fig. 3a).

### Cell viability assay

**Fig. 4:**
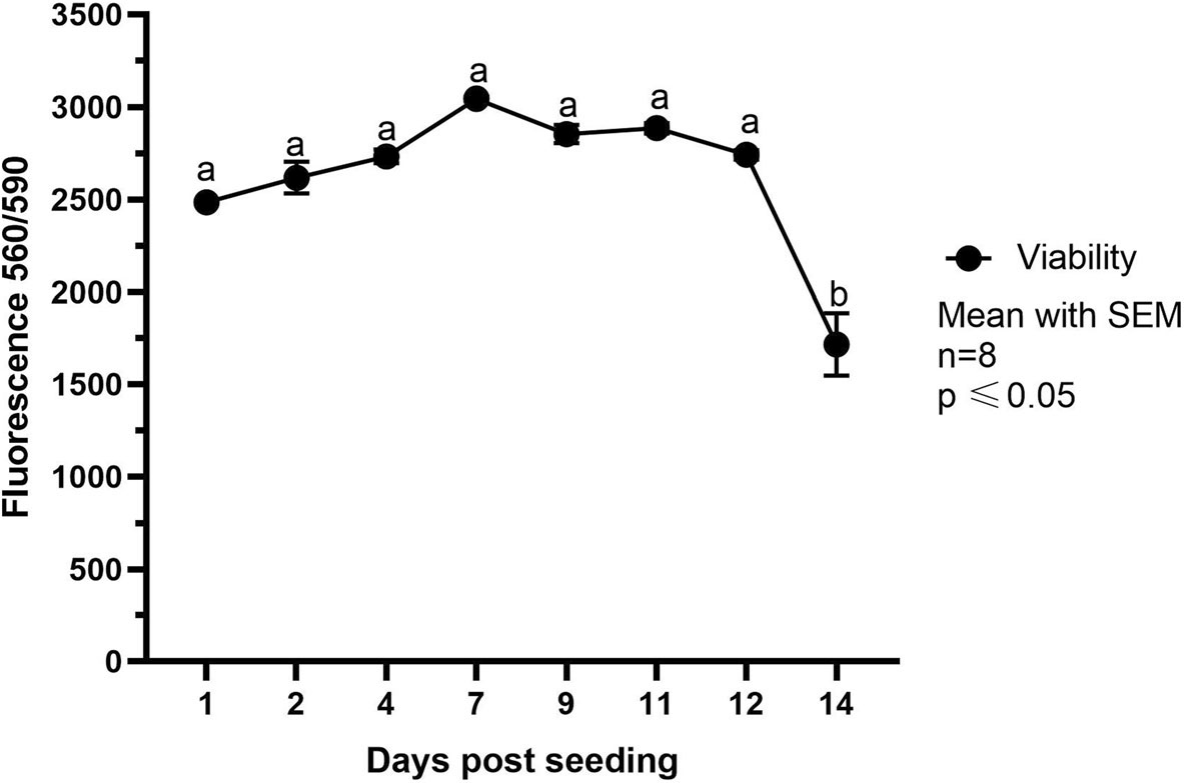
PrestoBlue® viability reported as fluorescence units (excitation 560 nm; emission 590 nm). Data are represented as Mean with SEM (*n* = 8). Each time point was compared with the previous one. Letters represent statistically significant (*p* ≤ 0.05) differences (one-way ANOVA Tukey’s multiple comparisons)

Cell viability was tested on days 1, 2, 4, 7, 9, 11, 12 and 14 after seeding. cIECs demonstrated a constant viability value for the first 12 days, as showed in Fig. 4 (Fig. 4). Afterward, viability dropped on day 14, corresponding to the time of appearance of holes in the monolayer (Fig. 1f). Each time point was compared with the previous one and no significant difference was detected within the first 12 days.

### RT- PCR characterization

**Fig. 5:**
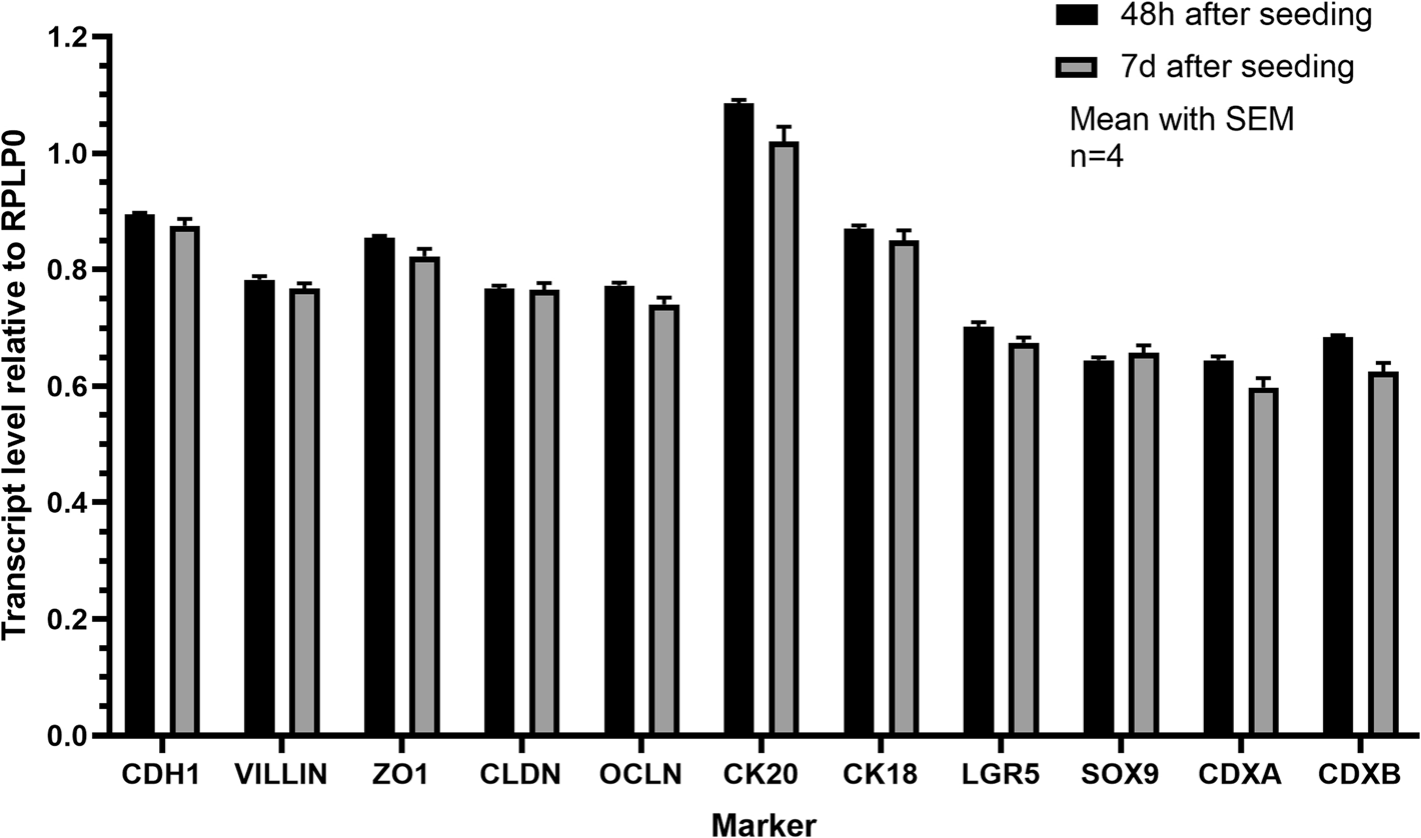
Expression of epithelial markers on cIECs at 48 h and 7d post-seeding. CDH1 = E-cadherin; ZO1 = Zonula occludens 1; CLDN = claudin 1; OCLN = occludin; CK20 = Cytokeratin 20; CK18 = Cytokeratin 18; LGR5 = Leucine-rich repeat-containing G-protein coupled receptor 5. Data are reported as transcript level normalized to RPLP0. No significant differences are present between the two time points within each marker (t-test). Data are represented as mean with SEM (*n* = 4)

cIECs were positive for both epithelial (*CHD1, VILL, CK18, CK20, ZO1, OCCL, CLDN)* and staminal markers (*LGR5, SOX9, CDXA,* and *CDXB*), confirming the presence of both differentiated and staminal cells in culture, as showed in Fig. 5 (Fig. 5). To assess the absence of contaminating endothelial cells, several markers typical of fibroblasts or mesenchymal cells were also tested, but none were detected (see Additional file 1). Differences in transcript levels between 48 h and 7 days post-seeding were not statistically significant (Fig. 5).

**Fig. 6:**
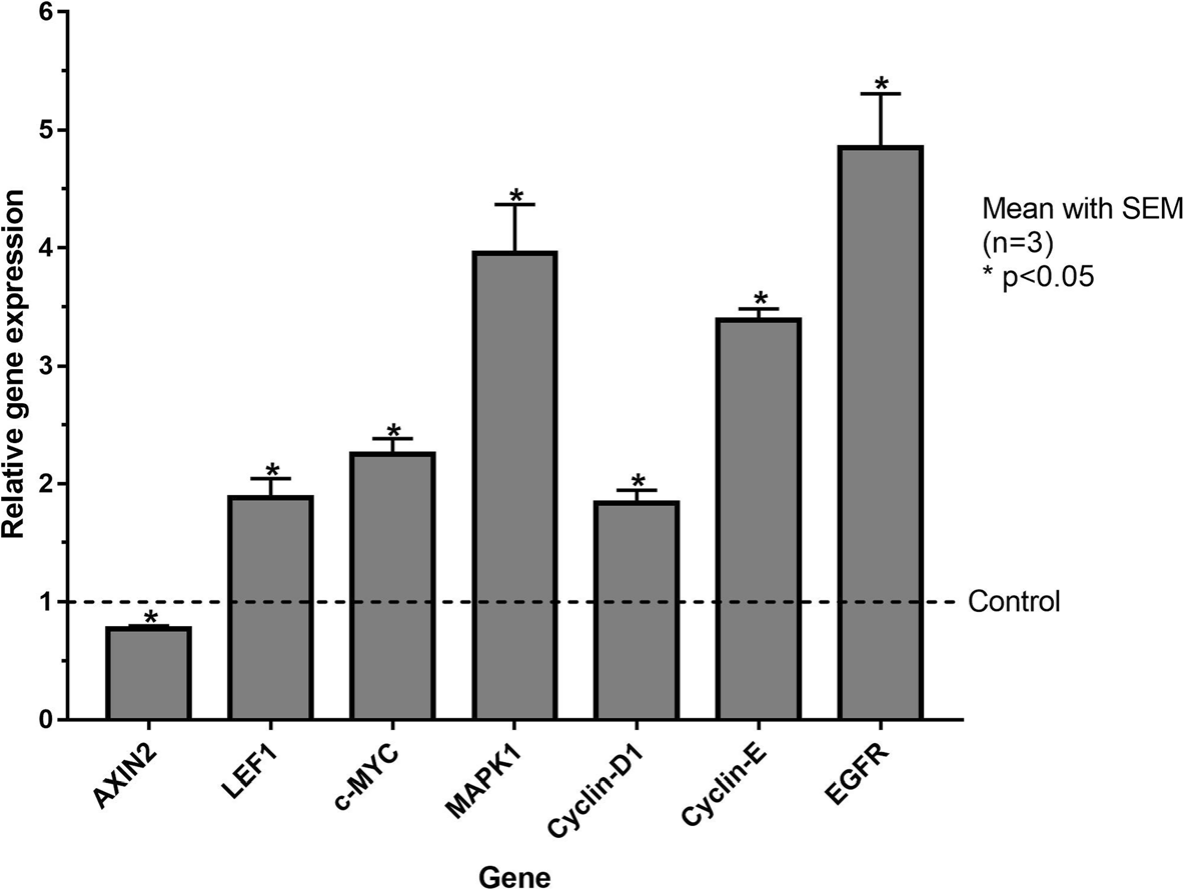
Expression of WNT and EGF pathways markers in cIECs cultured with (GF) and without (CTR) growth factors. AXIN2, LEF1 = Lymphoid Enhancer Binding Factor 1; c-Myc = MYC proto-oncogene bHLH transcription factor; MAPK1 = Mitogen-activated protein kinase 1; EGFR = Epidermal Growth Factor Receptor. Data are reported as 2^−DDCt^ values calculated by Delta–Delta Ct (DDCt) method. Expression was normalized to RPLP0, and data are represented as means ± SEM (*n* = 3) relative to the CTR group (represented with a dashed line). Significant differences between CTR and GF group (t-test) are represented as * (*p* < 0.05)

Furthermore***,*** cIECs cultured with R-spondin1, Noggin, CHIR99021, and hEGF showed a statistically significant expression modulation of transcription factors involved in cell proliferation downstream of beta-catenin/Wnt pathway and EGF pathway (AXIN2, LEF1, c-Myc, MAPK1, cyclin-D1, cyclin-E) and EGFR proving the efficacy of these factors to stimulate WNT and EGF proliferative pathways, as showed in Fig. 6 (Fig. 6).

### Immunofluorescence characterization

**Fig. 7:**
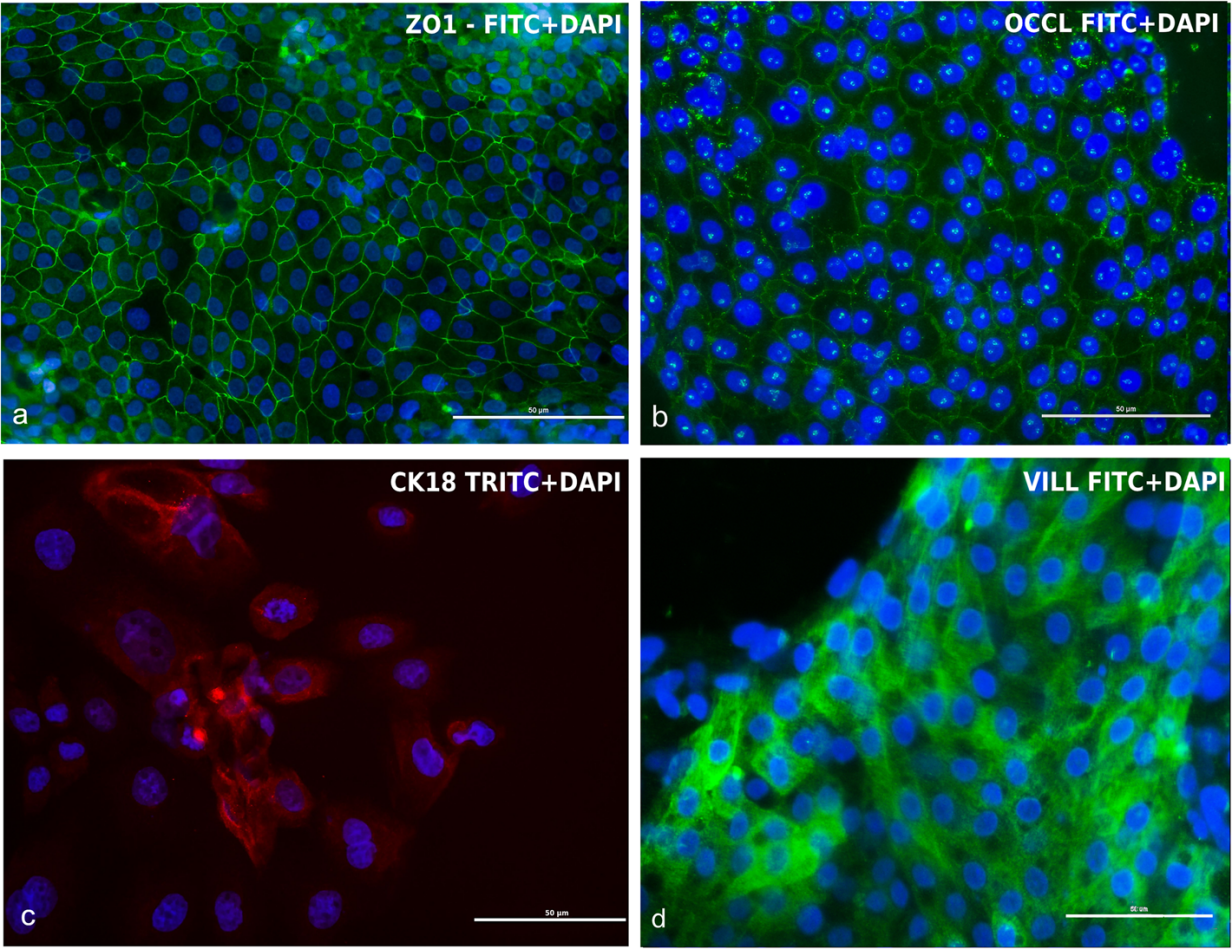
cIECs (7d after seeding) characterization by IF. **a** ZO1 (FITC+DAPI); **b** OCCL (FITC+DAPI); **c** CK18 (TRITC+DAPI); **d** Villin (FITC+DAPI). Cells were 100% positive (counted in three fields) for ZO1, OCCL, 97% for VILL, and 92% positive for CK18

**Fig. 8:**
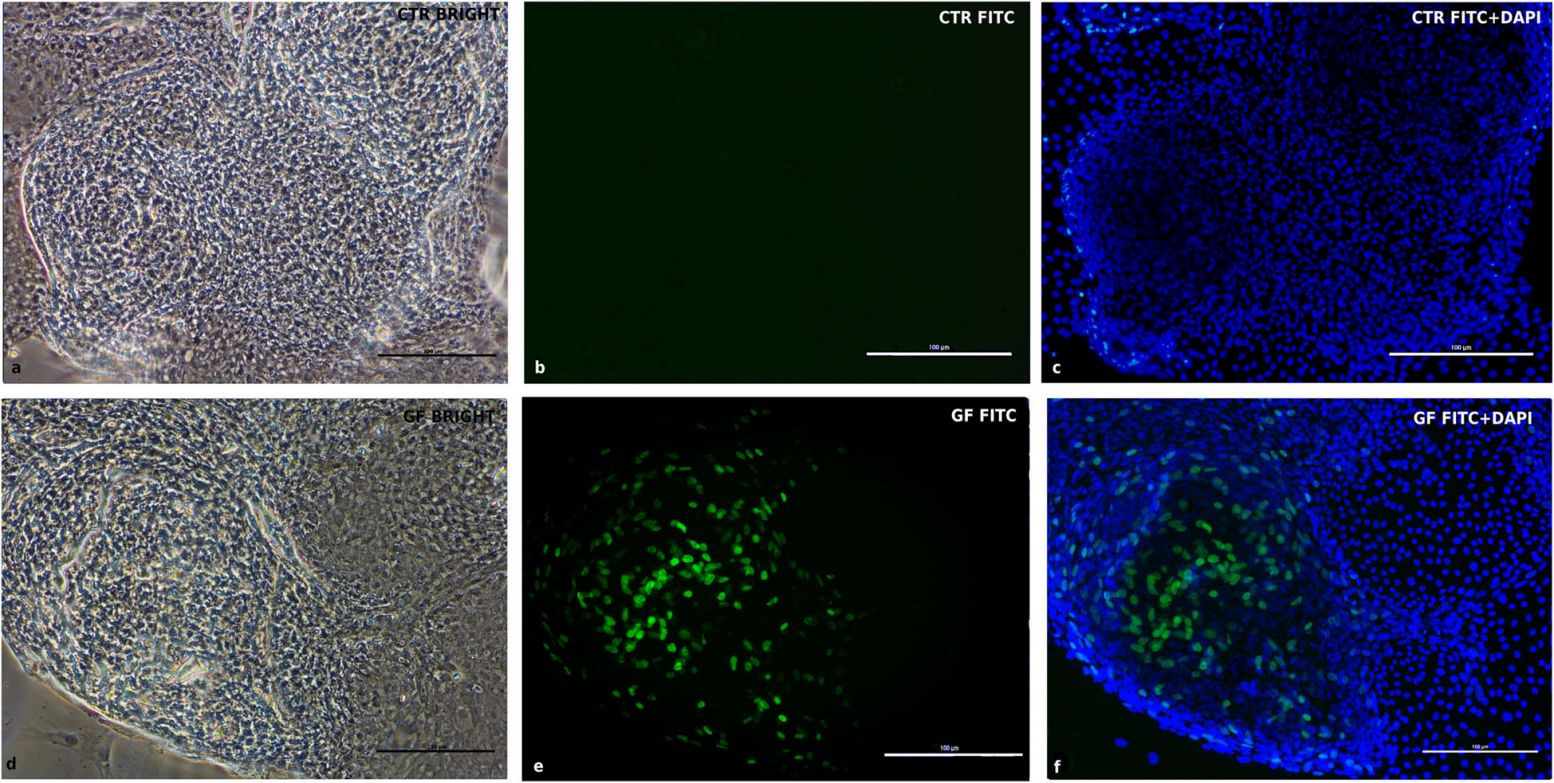
EdU staining at 5d after seeding. CTR = cIECs cultured without growth factors - GF = cIECs cultured with growth factors **a** CTR contrast-phase image; **b** CTR FITC only; **c** CTR FITC+DAPI; **d** GF contrast-phase image; **e** GF FITC only; **f** GF FITC+DAPI. The growth factors cocktail used can maintain in culture a transient amplifying cell population in active proliferation (represented by green nuclei in the picture). With complete differentiation, enterocytes lose this proliferative capacity

Confluent cells were stained 7 days after seeding for phenotype characterization. cIECs were 100% positive for ZO1 and OCCL, 92% for CK18, and 97% for VILL (Fig. 7), confirming the epithelial nature of these cells, as showed in Fig. 7. Furthermore, EdU incorporation, reported in Fig. 8, confirmed the presence of active proliferative centers in culture after 5 days from isolation (Fig. 8).

**Fig. 9:**
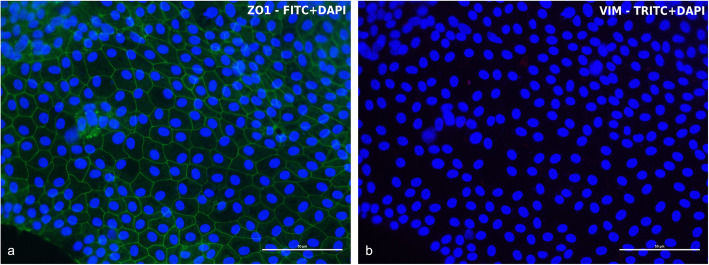
ZO1 and VIM expression by double staining IF. In this reaction the same sample was stained with two primary antibodies (ZO1 and VIM) at the same time, to prove the absence of VIM expression in ZO1 positive cIECs (11d after seeding). **a)** ZO1 (FITC+DAPI - 100% positive); **b** VIM (TRITC+DAPI - negative)

**Fig. 10:**
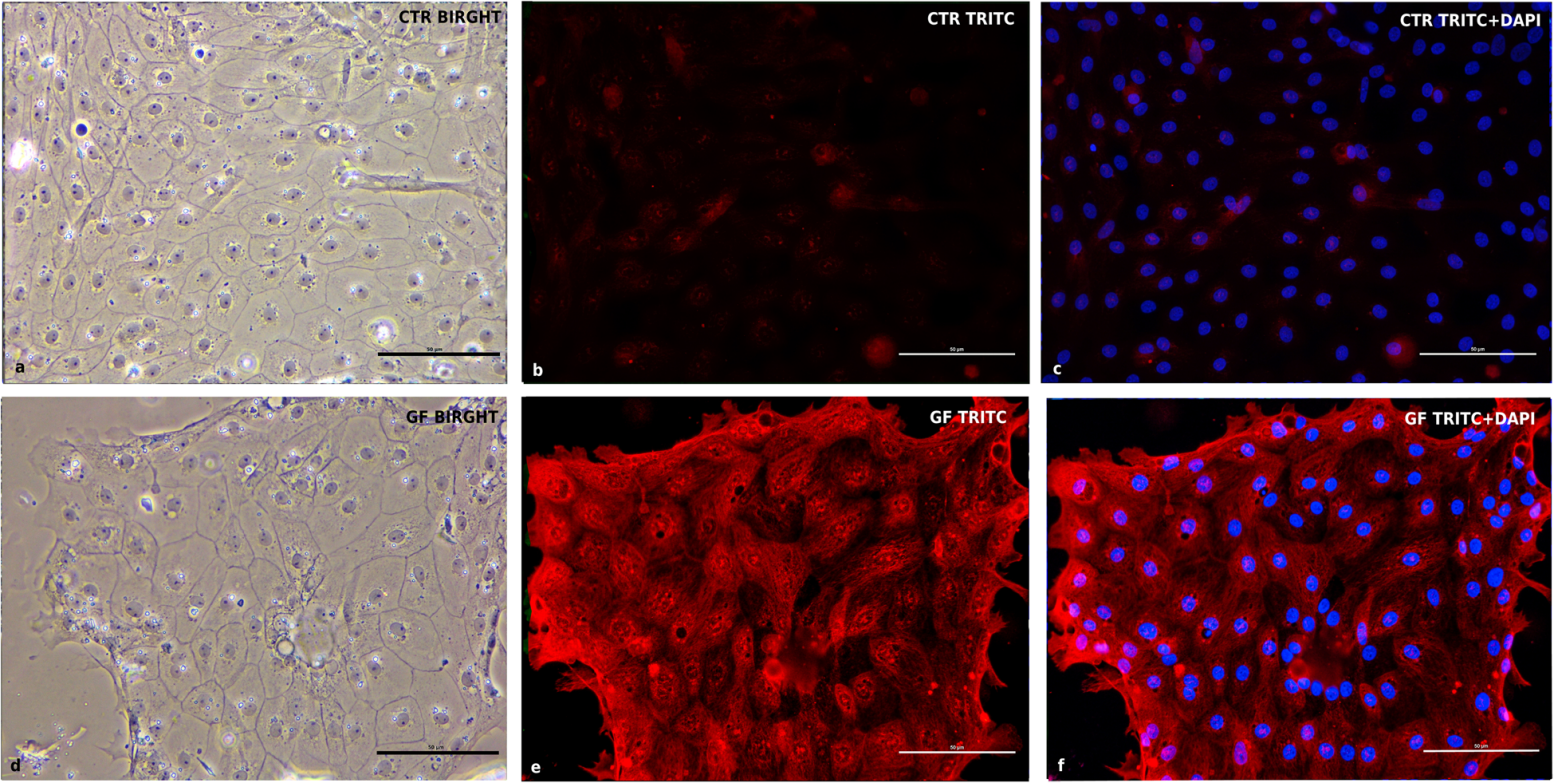
c-MYC expression by IF double staining 5d after seeding. CTR = cIECs cultured without growth factors - GF = cIECs cultured with growth factors **a** CTR contrast-phase image; **b** CTR TRITC only; **c** CTR TRITC+DAPI; **d** GF contrast-phase image; **e** GF TRITC only; **f** GF TRITC+DAPI. Cells were double-stained for c-MYC and MAPK1

**Fig. 11:**
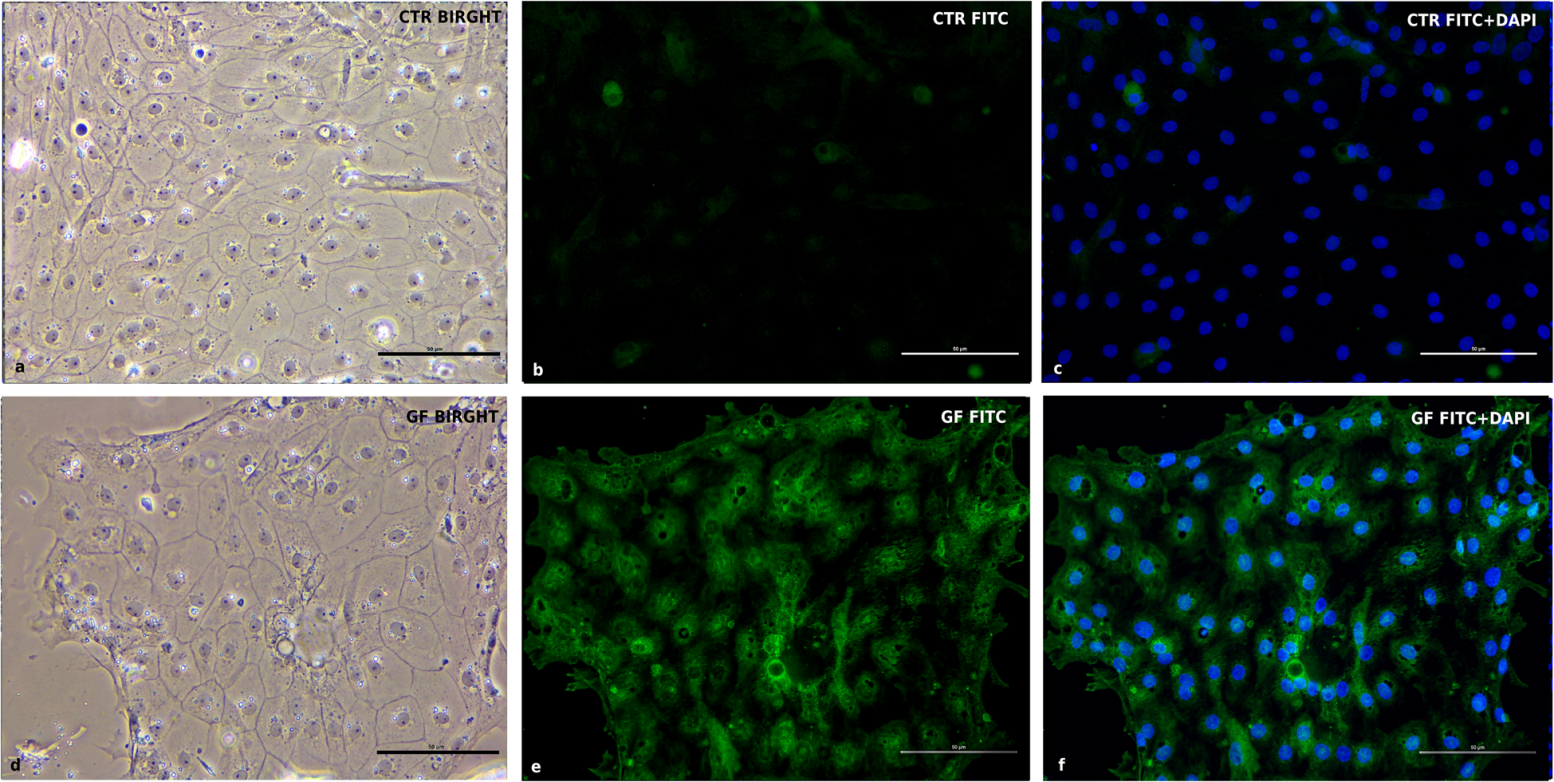
MAPK1 expression by IF double staining 5d after seeding. CTR = cIECs cultured without growth factors - GF = cIECs cultured with growth factors **a** CTR contrast-phase image; **b** CTR FITC only; **c** CTR FITC+DAPI; **d** GF contrast-phase image; **e** GF FITC only; f) GF FITC+DAPI. Cells were double-stained for c-MYC and MAPK1

To assess the absence of contaminating fibroblasts and endothelial cells, Vimentin (VIM), an intermediate filament protein expressed in these cell types, was marked, in Fig. 9, in double staining with ZO1 (Fig. 9). In this reaction the same sample was stained with two primary antibodies (ZO1 and VIM) at the same time, to prove the absence of VIM expression in ZO1 positive cells. cIECs appeared to remain negative at 11 days after seeding for VIM (Fig. 9b) but 100% positive for ZO1 (Fig. 9a). To have a positive control for VIM antibody, contaminating cells (that contain fibroblasts, myofibroblasts, and endothelial cells), isolated chicken embryos were used, recovering the < 40 μm fraction using the cIECs isolation procedure (Fig. 2). Lastly, IF double staining for c-MYC (Fig. 10) and MAPK (Fig. 11) proved the effective activation of the proliferative WNT and EGF pathway by the growth factors cocktail used, as showed in Fig. 10 and Fig. 11.

### Assessment of cIECs’ polarization capability

**Fig. 12:**
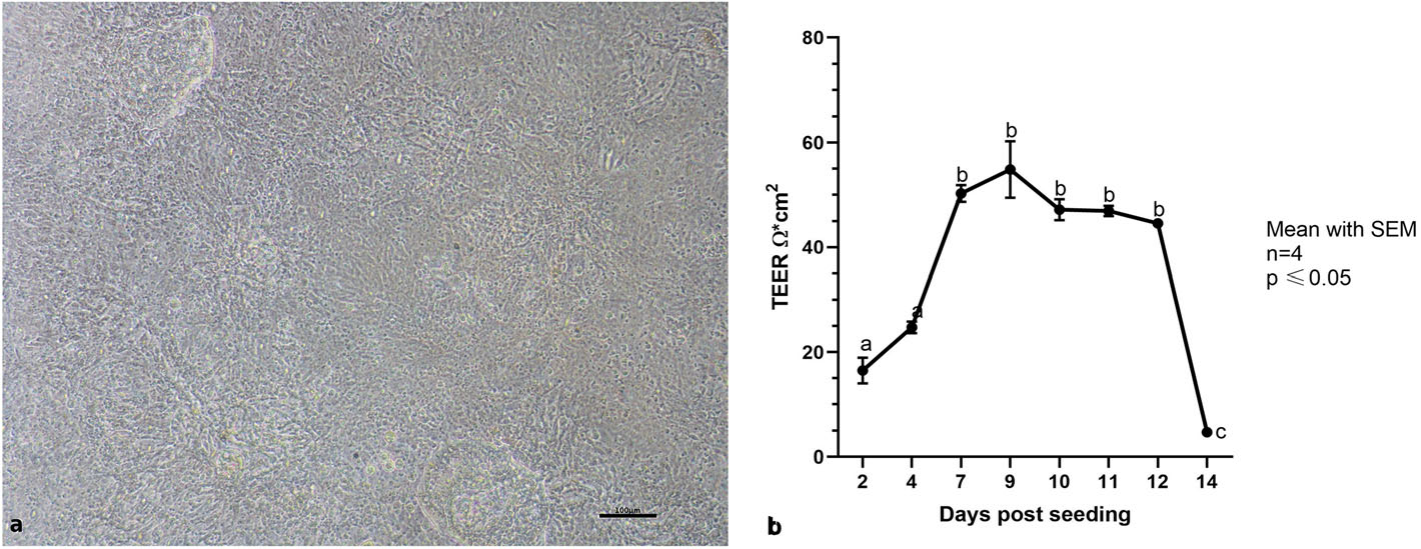
**a** cIECs cultured on Matrigel® matrix (2.5 mg/mL) coated Transwells® (7d after seeding) – **b** TEER values measured for 14 days in culture expressed as Ω*cm2 subtracted by blank. Mean with SEM (*n* = 4). Letters represent statistically significant (*p* ≤ 0.05) differences (one-way ANOVA Tukey’s multiple comparisons)

cIECs were grown on 0.4 μm Transwell® supports for 14 days (Fig. 12a) and the functional integrity of the monolayer was investigated. TEER gradually increased from 10 to 64 Ω*cm^2^ (Fig. 12b) and remained constant until day 12, as reported in Fig. 12.

## Discussion

The lack of an in vitro model of chicken intestinal cells is one of the most important limitations in applied poultry nutritional research [11]. In this study it has been developed a novel advantageous method to culture cIECs starting from chicken embryos. The major novelties compared to the methods currently available can be summarized as follows: enzyme mix of collagenase and hyaluronidase used during the isolation itself enabled to improve the yields of intestinal cell aggregates and a lower contamination of unwanted cells. The use of Matrigel®, a matrix that provides substrates for the cells to grow, allowed an improved attachment and expansion of the intestinal crypts; lastly, the use of different media specific for each stage provided an environment that allowed cells to expand, get clear of contaminating cells, express typical junctional proteins, and markers, and be viable until 12 days of culture. Finally, this is the first time that cIECs were cultured in Transwell® support and it is the first time that TEER measurement was reported. This is indeed a pre-requisite for studies involving epithelial integrity assessment.

Primary chicken cells, organoids, and immortalized mammalian cell lines are currently the only available possibilities for in vitro studies in applied intestinal poultry research [14]. 3D organoids are the in vitro model that can more closely represent the in vivo situation [15]. Intestinal organoids are self-organized 3D structures that reproduce the major characteristics of native tissue, showing also a folded epithelial morphology consisting of crypts and villi [16]. In 2012 Pierzchalska et al. [17], and later other research groups [18–20], reported the successful isolation of chicken organoids starting from 18-days-old embryos [17]. Unfortunately, their heterogeneity in terms of viability and shape limits the bioactive compounds penetration and they are usually unsuitable for feed additives screening and permeability studies, so monolayer cultures are needed [21, 22]. Primary cultures are also close to the in vivo intestinal epithelium than immortalized cell lines, but they are more difficult and expensive to obtain and maintain. Moreover, most of the isolation protocols for primary intestinal cultures are designed for mammalian species, but these methods cannot be easily shifted from one species to another [8, 23]. For this reason, a few isolation protocols for cIECs have been reported and there are no studies where these cells were able to become confluent maintaining the proper morphology and allowing the TEER measurement. In 2004 Immerseel and colleagues [5] created, for the first time, a primary chicken colonocyte culture, able to be infected by *S. enteritidis*, starting from adult chickens. Their cells were positive for cytokeratin in IF and showed the presence of microvilli on scanning electron microscopy but they survived only 3 days in culture [5]. Dimier-Poisson et al. (2004) [6] created a cIECs culture able to be infected by *E. tenella* and positive for CDH1 and cytokeratin on flow cytometry analysis, starting from chicken 18-days-old embryos. No data about survivability were present [6]. Later, Yuan et al. (2015) [10] obtained cIECs starting from 14-days-old embryos using the enzyme collagenase type I to recover intestinal aggregates. These were morphologically similar to the mouse intestinal crypts isolated by Ren and colleagues [24] and when seeded on coated plastic, they were able to generate a monolayer, that survived until 9d in culture; however, a clear cell characterization was missing. Both Kaiser et al. (2017) [11] and, more recently, Bai et al. (2019) [25] obtained cIECs with a proper morphology but those degenerated after 7-10d in culture. Kaiser et al. also compared monolayers isolated from embryonic and adult intestines, demonstrating no difference in growth. Despite that, the excessive mucus production of adult tissues makes them less suitable than embryonic ones for cIECs isolation. Moreover, Bar Shira and Friedman (2018) [12] isolated cIECs starting from 17-days-old embryos obtaining a culture positive for VILL and CDH1. They demonstrated that these cells can uptake and process bacteria, respond to bacterial products (LPS and LTA), and they express pro-inflammatory cytokine genes (interleukin 6, and 18) as well as acute-phase proteins avidin, lysozyme, and the secretory component derived from the polymeric immunoglobulin receptor. Nevertheless, no data about survivability, cIECs expansion, TEER o paracellular permeability were present [12]. Rath et al. (2018) [13] proved that it is also possible to create a cIECs line from intestinal tissues of adult chickens and to maintain these cells until 6–7 passages. However, those cells lack proper epithelial morphology since the IF characterization showed that ZO1 was not located in intercellular junctions, a typical tract of enterocytes [26]. Moreover, the usage of adult chickens requires, in the EU, a specific ethical authorization by the local animal care committee, which is a limitation for the possible applications.

In the present study, it was demonstrated that it is possible to improve primary cIECs expansion and survivability until day 12, using different cocktails of growth factors with specific timing. As reported by Thorne et al. (2018) [27], a Matrigel®-coated surface and a medium supplemented with R-spondin1, Noggin and hEGF, allowed to create a mouse enteroid monolayer expanded from intestinal crypts. This one was capable of self-renewing, polarizing, and generating major intestinal cell types, surviving for more than 2 weeks [27]. These factors and their receptors are largely conserved among different species, so they can potentially improve the renewal properties and survivability of cIECs in culture [18]. Developing different time-based media that contain these growth factors, a stable monolayer that can remain fully vital for more than 12 days was created, as proven by the viability assay. During the first 48 h, the key effector molecule was sodium butyrate that can promote the absorptive lineage of the intestinal stem cells in freshly isolated cell aggregates. Then, for the following days, the activation of the canonical beta-catenin/Wnt pathway was induced by R-spondin 1, Noggin, and CHIR99021 (GSK3 inhibitor), as already tested on chicken organoids by Li et al., (2019) [19]. Also, the EGF pathway was triggered by hEGF, and consequently, active proliferating centers positive to EdU staining were maintained. The actual activation of the cited pathways was confirmed by the upregulation of LEF1 induced by c-MYC as demonstrated by Hao et al. (2019) [28]. The downregulation of AXIN2 is another sign of WNT pathway activation as reported by Jho et al. (2001) [29]. Moreover, also the 5-fold increase of EGFR expression should be proof of EGF pathway activation. Lastly, the upregulation of other genes involved in cell proliferation such as MAPK1, Cyclin-D1, and Cyclin-E should be further evidence of the medium’s proliferation stimulation capability.

Thanks to reverse filtration, the usage of low FBS percentage, and the presence of heparin in the culture media, it was possible to block contaminating cell proliferation [30]. This was confirmed by comparing the adhesion and proliferation capacity of these cells cultured in a medium supplemented with heparin + 2% FBS or in a medium without heparin + 10% FBS, by the absence of positive cells for VIM in IF (tested at 7 and 11 days after seeding), as well as by the absence of several fibroblastic and mesenchymal markers during gene expression analysis. The presence of Villin (97%), CK18 (92%), the 100% positivity for ZO1, and OCCLN and proper localization of tight junctional proteins, confirmed the epithelial nature of these cells. Moreover, it was shown that cIECs can be cultured on Transwell® supports allowing TEER measurement that it has never been reported in the literature before, to our knowledge. TEER is a widely accepted quantitative technique to measure the integrity of tight junction dynamics in cell culture models [31]. cIECs, after 9d in culture on Transwells®, reached a TEER of 64 Ω*cm^2^, a value that is in line with other small intestinal cell lines, that range between 50 and 100 Ω*cm^2^ [32, 33]. Lastly, the molecular characterization also highlighted that the cellular composition of the isolated cIECs remained stable over time.

## Conclusions

In this study, we developed a novel method to culture cIECs, starting from chicken embryos and developing specific time-based media with specific key effector molecules.

These data suggest that cIECs, isolated and cultured with the novel protocol reported in this study, can become a suitable in vitro model for intestinal pathologies where enterocytes are involved. Further studies will be focused on the possibility to use cIECs as a model for applied research in poultry.

## Methods

### Animal care and usage

Specific pathogen-free (SPF) eggs were purchased from Charles River (Wilmington, Massachusetts, USA) and incubated in a semi-automated incubator at “Istituto Zooprofilattico Sperimentale della Lombardia e Emilia Romagna, sede territoriale di Forlì”. Chicken embryos were humanely euthanized by decapitation. On the 19th day of incubation, according to the AVMA guidelines and animal welfare, chick embryos were sacrificed by decapitation. As chick embryos older than 14 days can experience pain, decapitation was recommended as a humane method of euthanasia. According to the Italian legislation (D.lgs. 26/2014, the act on the protection of animals used for scientific and educational purposes, which was passed in March 2014 and transposed Directive 2010/63/EU into current Italian legislation), avian embryos are not considered as “live vertebrate animals”, so the approval of Animal Ethics Commission was not required.

### Intestinal epithelial cells isolation and culture

cIECs were isolated from 19-day-old SPF chicken embryos and intestines were recovered and placed in ice-cold hank’s balanced salt solution without magnesium and chloride (HBSS - Cat.# 55021C - Sigma-Aldrich, St. Louis, Missouri, USA), then they were cleaned from mesenteric fat and external mucus. The duodenal tract was harvested, opened longitudinally, cut into 1–2 mm pieces, and washed in ice-cold HBSS. Tissues were digested for 30 min at 37 °C with a digestion medium prepared as indicated in Table 1.

**Table 1:** Recipes of different media used during isolation protocol

Media	Composition
Digestion medium	• Dulbecco modified eagle’s medium high glucose (Cat.# D1145 - Sigma-Aldrich St. Louis, Missouri, USA);
	• 1% fetal bovine serum (Cat. # F7524 - Sigma-Aldrich, St. Louis, Missouri, USA).
	• 200 U/mL collagenase (Cat. # C9407 - Sigma-Aldrich, St. Louis, Missouri, USA).
	• 100 U/mL hyaluronidase (Cat. # H3506 - Sigma-Aldrich, St. Louis, Missouri, USA).
	• 1x penicillin-streptomycin (Cat. # P4333 - Sigma-Aldrich, St. Louis, Missouri, USA).
Washing medium	• Dulbecco modified eagle’s medium high glucose.
	• 1% fetal bovine serum;
	• 2% w/v sorbitol (Cat. # S1876 - Sigma-Aldrich, St. Louis, Missouri, USA).
	• 1x penicillin-streptomycin.
Basal medium	• Dulbecco modified eagle’s medium high glucose (DMEM).
	• 2% fetal bovine serum;
	• 1x penicillin-streptomycin;
	• 10 mM L-glutamine (Cat. # G7513 - Sigma-Aldrich, St. Louis, Missouri, USA).
	• 10 μg/mL insulin (Cat. # I6634 - Sigma-Aldrich, St. Louis, Missouri, USA).
	• 25 ng/mL human epidermal growth factor (Cat. # SRP3196 - Sigma-Aldrich, St. Louis, Missouri, USA)
	• 1 mM sodium pyruvate (Cat. # P5280 - Sigma-Aldrich, St. Louis, Missouri, USA).
	• 0.1 mg/mL heparin sodium salt from intestinal porcine mucosa (Cat. # H3149 - Sigma-Aldrich, St. Louis, Missouri, USA).
	• 1x non-essential amino acids (VWR, www.wvr.com– Cat.# X0557);
	• 1 mM sodium butyrate (Cat. # B103500 - Sigma-Aldrich, St. Louis, Missouri, USA).
	• 0,081 mg/L putrescine (Cat. # P5780 - Sigma-Aldrich, St. Louis, Missouri, USA).
	• 10 mM 4-(2-hydroxyethyl)-1-piperazineethanesulfonic acid) (HEPES - Cat. # H4034 - Sigma-Aldrich, St. Louis, Missouri, USA).

The digested tissue was centrifuged at 100 *g* for 3 min and resuspended in 37 °C washing medium (Table 1). The pellet was resuspended and filtered through a 100 μm cell-strainer (Cat.# 734–2762 - VWR, Radnor, Pennsylvania, USA) and then reversely filtered using a 40 μm cell-strainer (Cat.# 734–2760 - VWR, Radnor, Pennsylvania, USA) to recover the > 40 μm fraction. The recovered aggregates were then resuspended in basal medium (Table 1). They were ultimately plated in 24-wells (Cat.# 353,047 - Corning Incorporated, Corning, New York, USA) coated with Matrigel® matrix (Cat.# 356,234 - Corning Incorporated, Corning, New York, USA) diluted at 0.8 mg/mL with a density of 1000 aggregates/well or onto Transwell® (Cat.# 3495 - Corning Incorporated, Corning, New York, USA) supports with a pore size of 0.4 μm coated with Matrigel® (2.5 mg/mL). Cells were grown at 37 °C in a humidified atmosphere containing 5% CO_2_ and 95% air, as already studied by Kaiser et al., (2017) [11]. After 24 h, the presence of cell clusters and the degree of endothelial cell contamination was assessed through observation under an inverted phase-contrast microscope (Nikon Eclipse TS100 - www.nikon.com). Then the culture was washed with HBSS to remove unattached and dead cells and the foci of proliferating enterocytes were replenished with fresh medium. To improve cIECs survivability different growth factors were supplemented to the basal medium. In particular, for the first 48 h, the key effector molecule was sodium butyrate, then, for the following days, the activation of canonical beta-catenin/Wnt pathway was induced by adding R-spondin 1, Noggin, and CHIR99021 (GSK3 inhibitor) and EGF pathway was triggered by human epidermal growth factor (hEGF – it was used due to the low chicken EGF availability). Table 2 reports the combination of the different concentrations of growth factors used according to the different time-points after seeding.

**Table 2:** Recipes of different media based on time post-seeding

	BUTYRATE	R-SPONDIN 1	NOGGIN	hEGF	Y-27632	CHIR99021
First 48 h	YES (1 mM)	NO	NO	YES (25 ng/mL)	YES (10 μM)	NO
After 48 h	NO	YES (500 ng/mL)	YES (100 ng/mL)	YES (50 ng/mL)	NO	YES (3 μM)

After the first 48 h, the medium change was performed every other day. These supplements were added to the basal medium. R-spondin1 (Cat.# SRP3292 - Sigma-Aldrich, St. Louis, Missouri, USA), Noggin (Cat.# 21–7075-U020 - Tonbobio, San Diego, California, USA), CHIR99021 (Cat.# SML1046 - Sigma-Aldrich, St. Louis, Missouri, USA), Y-27632 (Cat.# Y0503 - Sigma-Aldrich, St. Louis, Missouri, USA)

Cells have been passed using trypsin-EDTA 0.05% (Cat.# 25–052-CI- Corning Incorporated, Corning, New York, USA) or Accutase® (Cat.# 25–058-CI- Corning Incorporated, Corning, New York, USA) for 5′ at 37 °C and seeded on new coated well at 3.5 × 10^5^ cell/cm^2^.

### Contaminating cell removal

Contaminating cells (fibroblasts, myofibroblasts, and endothelial cells) were obtained using chicken embryos with the same cIECs isolation protocol and recovering the < 40 μm fraction from reverse filtration. These cells, differently from cIECs, are plastic adherent so they were cultured on non-coated plastic flasks and glass slides using as culture medium: Dulbecco modified eagle’s medium high glucose (DMEM) with 10% fetal bovine serum (FBS), 1x penicillin-streptomycin, 1x non-essential amino acids and 10 mM L-glutamine at 37 °C in a humidified atmosphere containing 5% CO_2_ and 95% air. To prove the efficacy of heparin sodium salt and reduced FBS in preventing contaminating cell adhesion, when confluent, they were divided into two groups and seeded on coated wells with Matrigel® matrix diluted at 0.8 mg/mL. Both groups were seeded on Matrigel® coated wells to eliminate the doubt that Matrigel® matrix could prevent adhesion of contaminating cells by releasing small amounts of heparin into the medium. Then one group received the same medium (CTR) and the other one received the medium added with 0.1 mg/mL heparin sodium salt from intestinal porcine mucosa and reduced FBS (2%) (HEPA+FBS2).

### Cell viability assay

cIECs viability was assessed with PrestoBlue® fluorescent cell viability reagent (Cat. # A13262 - Thermo Fisher Scientific, Waltham, Massachusetts, USA), according to the manufacturer’s protocol. PrestoBlue® is a resazurin-based reagent that works as a cell viability indicator by using the reducing power of living cells. Resazurin is blue in color and nonfluorescent and, when added to cells, it is reduced in the mitochondria of metabolically active cells to resofurin, which is pink in color and highly fluorescent. Highly viable cells produce high fluorescence. Cells were cultured on Matrigel®-coated 96-well (Cat.# 353,072 - Corning Incorporated, Corning, New York, USA) using white Dulbecco’s modified Eagle medium (DMEM - Cat.# D1145 - Sigma-Aldrich, St. Louis, Missouri, USA) to avoid phenol red interference. They were incubated for 3 h with PrestoBlue®, then fluorescence was detected (excitation 560 nm; emission 590 nm) with Thermo Scientific Varioskan™ LUX multimode microplate reader (Cat. # VL0000D0 - Thermo Fisher Scientific, Waltham, Massachusetts, USA).

### Monolayer integrity

To evaluate the monolayer’s integrity and the presence of tight junctions, the measurement of transepithelial electrical resistance (TEER) was performed. Cells were cultured on Transwell® cell supports (pore size 0.4 μm) coated with Matrigel® matrix diluted at 1.5 mg/mL. TEER was measured with a volt-ohm meter (Millicell ERS-2, Cat.# MERS00002 – Millipore, Burlington, Massachusetts, USA) on day 2, day 4, day 7, day 9, day 10, day 11, day 12 and day 14. TEER values were calculated as [(*cell well TEER* − *blank well TEER*) × *well area size* = (*Ω* · *cm*2)].

### RT-PCR

RT-PCR characterization was performed for different epithelial, staminal, and non-epithelial markers. Total RNA was isolated using the NucleoSpin® RNA Kit (Cat. # 750955 - MACHEREY-NAGEL Inc., Bethlehem, USA) according to the manufacturer’s protocol. RNA yield and quality were determined spectrophotometrically detecting 260 and 280 nm absorbance by Denovix® DS-11 Series Spectrophotometer/Fluorometer (Microvolume Mode with Smart Path Technology – Denovix®, Hanby Building, Wilmington, USA). RNA was reverse transcribed with iScript® cDNA Synthesis Kit (Cat. # 1708890 - Bio-Rad Laboratories, Hercules, California, USA) according to the manufacturer’s instructions. Lastly, real-time PCR reactions were performed in duplicate using the CFX96 Touch Real-Time PCR Detection System and iTaq® Universal SYBR Green Supermix (Cat. # 1725120 - Bio-Rad Laboratories, Hercules, California, USA). Gene expression was reported as relative transcript level to 60S acidic ribosomal protein P0 (*RPLP0*), calculated as the mean Cq of each gene of interest divided by the mean Cq of RPLP0. For the characterization of cIECs, specific markers of epithelial intestinal cells were chosen, according to literature: E-cadherin (*CHD1*), villin (*VILL*), cytokeratin 18 (*CK18*), cytokeratin 20 (*CK20*), zonula occludens-1 (*ZO1*), occludin-1 (*OCCL*) and claudin-1 (*CLDN*). Markers typical of fibroblasts and mesenchymal cells were also selected to exclude the presence of these other cell types: cluster of differentiation (**CD**) 34, *CD45, CD146, CD90, CD73*, and *CD105*. As intestinal stem cell markers, leucine-rich repeat-containing G-protein coupled receptor 5 (*LGR5*), *SOX9, CDXA*, and *CDXB* (chicken homologous of human *CDX2*) were chosen.

To prove the activation of WNT and EGF pathways, different transcriptional factors were selected: AXIN2, lymphoid enhancer binding factor 1 (LEF1), MYC proto-oncogene bHLH transcription factor (c-MYC), mitogen-activated protein kinase 1 (MAPK1), cyclin-D1, and cyclin-E. Moreover, to prove the efficacy of hEGF on the EGF pathway, the effect on epidermal growth factor receptor (EGFR) expression was tested. For this purpose, two groups of freshly isolated cIECs have been used. For the first 48 h, the two groups received the same basal medium supplemented with butyrate, hEGF (25 ng/mL) and Y-27632 (Table 2, First 48 h medium). After 48 h, the first group received the same medium (Control group, CTR) and the second group received a basal medium supplemented with R-spondin1, Noggin, hEGF (50 ng/mL), and CHIR99021 (Growth factor group, GF – Table 2, After 48 h medium), then cells were cultured for 3 days. Cells treatments were stopped after 5 days because cIECs cultured without growth factors begin to die after 5 days in culture.

All primers (Sigma-Aldrich, St. Louis, Missouri, USA) were designed using the PrimerBLAST tool (https://www.ncbi.nlm.nih.gov/tools/primer-blast/) and they are listed in Table 3.

**Table 3:** Primer list used to characterize cIECs designed with PrimerBLAST

	*Gene*	*Primer sequence (5′- > 3′)*	*Product length (bp)*	*Accession N.*
Epithelial markers	E-cadherin	F: TGAAGACAGCCAAGGGCCTG R: CTGGCGGTGGAGAGTGTGAT	109	NM_001039258
	Villin	F: GAACCTCTCGTGGCACCGC R: CTCATGTCCCTGCACCTCCC	152	XM_418521.5
	Cytokeratin 18	F: CACAGATCCGGGAGAGCCTG R: CTCCACCGCGCTGTCATAGA	110	XM_025145666
	Cytokeratin 20	F: GCGCGTTATAAAGGAGGAGCTG R: CGCTGATTTACGGGCCGAAC	200	NM_204749.2
	Zonula occludens-1	F: TCTGCACAGTGAGGTTGGCT R: GGCTGTCCTGCATCGGTGT	145	XM_004934975
	Occludin-1	F: TGCTTTTGCCCAAGCAGGAA R: TGTGGGAGAGGCACCAGTTG	153	NM_204417
	Claudin-1	F: TCGGTGGTGGTCACTTCGTC R: CGCTGATTTACGGGCCGAAC	113	NM_001004768
Staminal markers	LGR5	F: TGGGCTCCACAGCCTAGAGA R: CCTACAAACGCACGCTCAGG	144	XM_425441.5
	SOX9	F: CCACCATGTCGGATGACTCC R: GGTGTTCTCTTGAGGTCGGG	86	NM_204281.1
	CDXA	F: CAGTGAGTGTCCCCCATGTC R: GGGACAGATGTCTGCAGGTC	92	NM_204676.2
	CDXB	F: ATCTGGTTCCAGAATCGCCG R: TGGTGGGAACAGGGAACTTG	141	NM_204614.1
Endothelial cell markers	CD34	F: TCTGCACAGTGAGGTTGGCT R: GGCTGTCCTGCATCGGTGT	153	XM_004934975
	CD45	F: TCCGACGCAGAGTGAATGCT R: AGCCCCTCCAACATAGCGTC	118	NM_204417.2
	CD146	F: TCACAAAGCCAGAGGCACGTA R: AGCAGGAAAGGGTCGAAGGA	196	NM_001004768
	CD90	F: GAACGTCTACCGGAACCGAG R: GTGTAGTCGTTGGTGGCCTT	126	NM_204381.2
	CD73	F: GGGCACTCTGGCTTTACTGT R: GGAGGGAGGGGTTCCTGTAT	111	XM_004940396
	CD105	F: GCGAAAATAGCAACAGCCCC R: GTACAGCCCTTCACCTCACG	103	NM_001080887
WNT and EGF pathways	AXIN2	F: ACCGAGACGTTGGAGAACGG R: ATCCGTCAGGGCATCACTGG	138	NM_204491.1
	c-MYC	F: CTGGTCCTCAAGCGGTGTCA R: GACCCTGCCACTGTCCAACT	114	XM_015283089
	LEF1	F: CCCACCCATCTCACATCCCC R: GTGAAAACCAGCCAAGAGGTGG	141	NM_205013.2
	MAPK-1	F: TGTGACTTCGGACTGGCTCG R: AGGAGCCCTGTACCAACGTG	93	XM_015275131
	Cyclin-D1	F: CGAGGTGGAGACCATCCGAC R: GAAGTAGGACACCGAGGGCG	112	NM_205381
	Cyclin-E	F: CGTTCCTCCAGGATCCCGAC R: CCAGGACAGCTGGTTTTCGT	80	NM_001031358
	EGFR	F: CCGCAGCTGGGTAGGCAC R: GCTCCTCACTCTGCACTGCT	129	NM_205497
Reference	RPLP0	F: TCACGGTAAAGAGGGGAGGTG R: CTTGCTCAGTCCCCAGCCTT	143	NM_205179

### Immunofluorescence

Immunofluorescence (IF) staining was performed for Cytokeratin 18, Zonula occludens 1, Occludin, Villin, and Vimentin to characterize cIECs. For the characterization freshly isolated cIECs were cultured for 7 or 11 days onto 10 mm Matrigel®-coated glass slides (0.8 mg/mL). Then an IF double staining was also performed for c-MYC and MAPK1 to prove the activation of WNT and EGF pathways. For this purpose, freshly isolated cIECs have been divided into two groups. For the first 48 h, the two groups received the same basal medium supplemented with butyrate, hEGF (25 ng/mL) and Y-27632 (Table 2, First 48 h medium). After 48 h, the first group (CTR) continued to receive the same medium while the second group (GF) received the basal medium supplemented with R-spondin1, Noggin, hEGF (50 ng/mL), and CHIR99021 (Table 2, After 48 h medium), then cells were cultured for 3 days. Cells treatments were stopped after 5 days because cIECs cultured without growth factors begin to die after 5 days in culture.

Generally, cells were fixed for 20 min with 4% paraformaldehyde (Cat.# 158,127 - Sigma-Aldrich, St. Louis, Missouri, USA) in Dulbecco’s phosphate buffered saline (DPBS - Cat.# D8537 - Sigma-Aldrich St. Louis, Missouri, USA). Cells were permeabilized with 0.5% Triton X-100 (Cat.# VARICP3418 - VWR, Radnor, Pennsylvania, USA) for 15 min and then blocked in 10% goat serum (Cat.# G9023 - Sigma-Aldrich St. Louis, Missouri, USA) for 1 h. Primary monoclonal antibodies were diluted in 2% bovine serum albumin (BSA - Cat.# P6154 - VWR, Radnor, Pennsylvania, USA) + 0.05% saponins (Cat.# A18820 - Alfa Aesar, Haverhill, Massachusetts, USA) in DPBS as reported in Table 4.

**Table 4:** Antibodies used for immunofluorescence characterization

Reagent	Dilution	Product catalog number
Mouse anti-chicken cytokeratin 18	10 μg/mL	MA1–06326
Rabbit anti-chicken zonula occludens 1	10 μg/mL	61–7300
Rabbit anti-chicken pan-occludin	2 μg/mL	71–1500
Mouse anti-chicken vimentin	2 μg/mL	MA5–11883
Rabbit anti-villin	10 μg/mL	ab130751
Rabbit anti-MAPK1/ERK2	1:100	MA5–15134
Mouse ant-c-Myc	10 μg/mL	MA1–16637
Donkey anti-mouse secondary antibody, TRITC conjugated	4 μg/mL	A16016
Goat anti-rabbit secondary antibody, FITC conjugated	4 μg/mL	A27034

All antibodies were purchased from Invitrogen (Thermo Fisher Scientific, Waltham, Massachusetts, USA) except anti-villin that was purchased from Abcam plc (Cambridge, UK)

Cells were incubated with primary antibodies for 3 h at + 4 °C in a humidified chamber. Secondary antibodies conjugated to fluorescein isothiocyanate (FITC) or tetramethylrhodamine (TRITC) were used to probe the bounded primary antibodies for 1 h (Table 4), followed by two successive washes with 0.2% BSA + 0.05% saponins in DPBS. The slides were then mounted with Fluoroshield® containing 4′,6-diamidine-2′-phenylindole dihydrochloride (DAPI) (Cat.# F6057 - Sigma-Aldrich, St. Louis, Missouri, USA). Images were acquired with Nikon Eclipse Ci fluorescence upright microscope (Nikon corporation - www.nikon.com) and processed with NIS-Elements® software (Nikon corporation - www.nikon.com). Percentages of positive cells were obtained evaluating the ratio between positive cells and total nuclei in three different fields.

### EdU proliferation assay

To assess the presence of proliferative centers in culture, 5-ethynyl-2′-deoxyuridine (EdU) incorporation, which can specifically mark the S-phase cells, was performed on the fifth day of culture. The EdU-Click 488 (Cat.# BCK-EDU488- Sigma-Aldrich, St. Louis, Missouri, USA) was used. Briefly, 4 h after the administration of 10 μM EdU in the culture medium, cells were then fixed in 4% paraformaldehyde for 20 min at room temperature. After permeabilizing with 0.5% Triton X-100 in PBS for 20 min and rinsing 3 times with 3% BSA in PBS, cells were observed under the fluorescence upright microscope. The EdU-positive cells then showed up as a green color. For this purpose, two groups of cIECs have been used (*n* = 4). For the first 48 h, the 2 groups received the same basal medium supplemented with butyrate, hEGF (25 ng/mL), and Y-27632 (Table 2, First 48 h medium). After 48 h, the first group (CTR) received the same medium while the second group (GF) received the basal medium supplemented with R-spondin1, Noggin, hEGF (50 ng/mL), and CHIR99021 (Table 2, After 48 h medium), then cells were cultured for 3 days. Cell treatments were stopped after 5 days because cIECs cultured without growth factors begin to die after 5 days in culture.

### Statistical analysis

For viability assay, TEER measurement and gene expression data were represented as mean ± standard error (SEM). Statistical analyses were performed using Graph-pad Prism® 8.3 (https://www.graphpad.com/scientific-software/prism/). Viability and TEER data were analyzed using one-way ANOVA with Tukey’s multiple comparison test and gene expression ones with a Student’s t-test. The level of significance was set at 0.05.

## Supplementary Information

**Additional file 1: **In the additional file the full characterization of cIECs by qPCR is reported, including the negative results for contaminating cell markers. Moreover, also a detailed description of the contaminating cell markers with a qPCR (positive control) made on contaminating cells recovered from the embryos, is reported.

## Data Availability

The datasets generated and/or analyzed during the current study are available from the corresponding author on reasonable request.

## Abbreviations

BSA: Bovine serum albumin
CD: Cluster of differentiation
CDH1: E-cadherin
cIECs: Chicken intestinal epithelial cells
CK18: Cytokeratin 18
CK20: Cytokeratin 20
CLDN: Claudin-1
c-MYC: MYC proto-oncogene bHLHtranscription factor
DAPI: 4’,6-diamidine-2’-phenylindole dihydrochloride
DMEM: Dulbecco modified eagle’s medium high glucose
DPBS: Dulbecco’s phosphate buffered saline
EdU: 5-ethynyl-2’-deoxyuridine
EGFR: Epidermal Growth Factor Receptor
FBS: Fetal bovine serum
FITC: Fluorescein isothiocyanate
HBSS: Hank's balanced salt solution without magnesium and chloride
hEGF: Human epidermal growth factor
IF: Immunofluorescence
LEF1: Lymphoid Enhancer Binding Factor 1
LGR5: Leucine-rich repeat-containing G-protein coupled receptor 5
MAPK1: Mitogen-activated protein kinase 1
OCCL: Occludin-1
RPLP0: 60S acidic ribosomal protein P0
SEM: Standard error
SPF: Specific pathogen-free
TEER: Transepithelial electrical resistance
TRITC: Tetramethyl rhodamine
VILL: Villin
VIM: Vimentin
ZO1: Zonula occludens-1
