# Supplementary material for: Isolation, culture, and characterization of chicken intestinal epithelial cells
Source: BMC Mol Cell Biol. 2021 Feb 12;22:12. doi: 10.1186/s12860-021-00349-7 (PMC7881477; doi:10.1186/s12860-021-00349-7)
Supplement: Supplementary file 1 — Additional file 1: In the additional file the full characterization of cIECs by qPCR is reported, including the negative results for contaminating cell markers. Moreover, also a detailed description of the contaminating cell markers with a qPCR (positive control) made on contaminating cells recovered from the embryos, is reported. [file 12860_2021_349_MOESM1_ESM.docx]

**ADDITIONAL FILE 1**


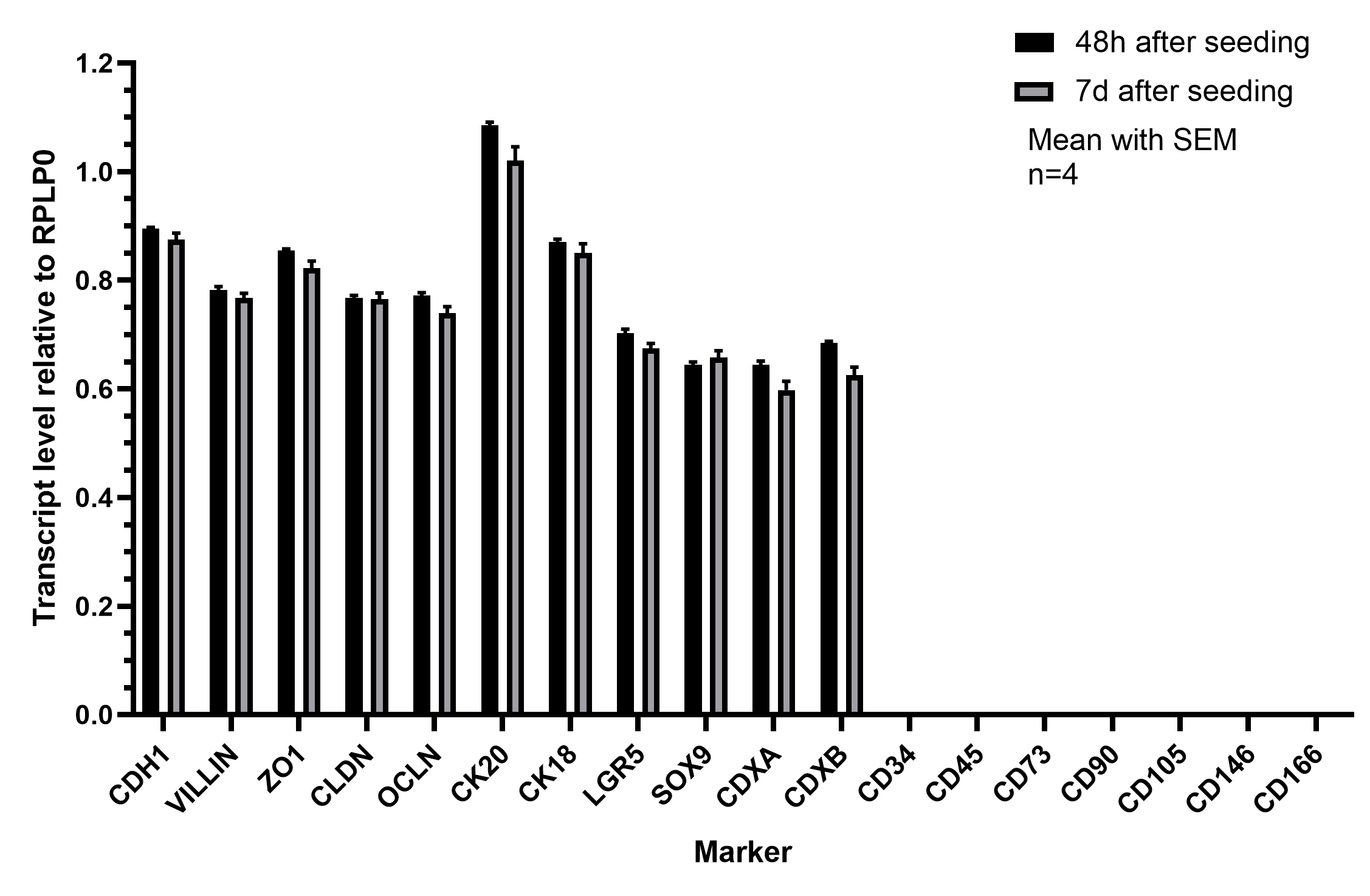


**Additional figure 1:** cIECs have been characterized for different epithelial and non-epithelial (contaminating) cell markers at 48h and 7d after seeding, to prove the purity of cells in culture. Data are reported as transcript level normalized for RPLP0. No significant differences are present between each the 2 time points for each marker (t-test). Data are represented as mean with SEM (n=4).


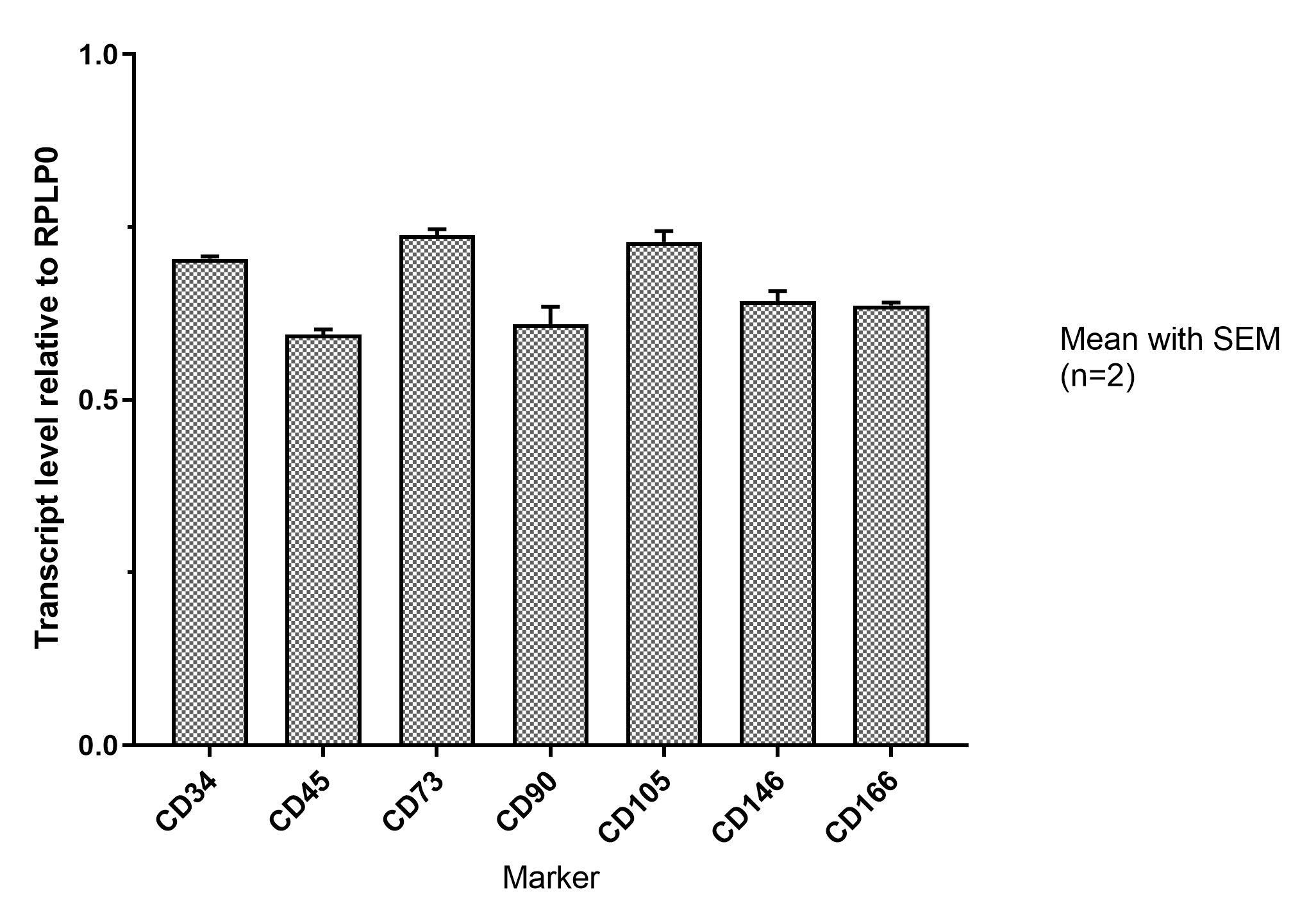


**Additional figure 2:** Contaminating cells have been characterized in RT-PCR for all different cell markers as positive control. Data are reported as transcript level normalized for RPLP0. Data are represented as mean with SEM (n=2).

**CDH1:** CDH1 is the subtype of cadherin protein expressed only by epithelial cells.

**VILLIN:** Villin is one of the actin binding proteins found in microvilli which compose brush borders. It is tissue-specific marker for intestinal cells (https://doi.org/10.1007/BF01349345).

**ZONULA OCCLUDENS 1:** Zonula occludens 1 is a multi-domain polypeptide required for the assembly of epithelial tight junctions (10.1242/jcs.113399)

**OCCLUDIN:** Occludin has been identified as an integral transmembrane protein localizing at the tight junction and directly associated with ZO-1, an undercoat-constitutive cytoplasmic protein.

**CLAUDIN1:** Claudins are tight junction integral membrane proteins that are key regulators of the paracellular pathway in the intestinal epithelium.

**CYTOKERATIN 20:** Keratin 20, a member of the intermediate filament and a well-known marker of intestinal differentiation (10.1073/pnas.0812904106).

**CYTOKERATIN 18:** KRT18 is the type I intermediate filament and together with its filament partner keratin 8, are the most found members of the intermediate filament gene family. They are expressed in single layer epithelial tissues.

**LGR5:** LGR5 is a biomarker of adult stem cells in intestinal epithelial tissue.

**SOX9:** SOX9 acts as a regulator of proliferation and differentiation in epithelial stem/progenitor cells in intestinal crypts (https://doi.org/10.2144/0000113851).

**CDXA:** CDXA is a homeobox gene (is a homolog of mammals' cdx2) of the caudal type that was shown to be expressed in the endoderm-derived gut epithelium during early embryogenesis. Expression of the CDXA protein was reported during intestine morphogenesis from 11 days of incubation to adulthood in the chicken in the epithelium of the villi (https://doi.org/10.2144/0000113851).

**CDXB:** The chicken CDXB expression pattern indicates that it is present in all intestinal epithelial cells (https://doi.org/10.2144/0000113851).

**CD34**: CD34 is a transmembrane phosphoglycoprotein, found in endothelial progenitor cells, a subset of mesenchymal stem cells, hematopoietic cells, and ECs of blood vessels and pleural lymphatic vessels. The presence of CD34 on nonhematopoietic cells in various tissues has been linked to progenitor and adult stem cell phenotypes.

**CD45**: Commonly used marker of hematopoietic cells.

**CD73**: CD73 is one of the classic markers that specifically define the mesenchymal stem cells population (https://doi.org/10.1155/2019/8717694)

**CD90**: CD90 has been identified on a variety of stem cells and at varying levels in non-lymphoid tissues such as on fibroblasts, brain cells, and activated endothelial cells and it has been used as a human primary cell biomarker to develop an efficient and reliable method for eliminating unwanted or contaminating fibroblasts from human primary cell cultures (10.1007/s10616-009-9190-3)

**CD105**: Endoglin (CD105) is a cell membrane glycoprotein predominantly expressed on cellular lineages within the vascular system, and over-expressed on proliferating endothelial cells (10.2174/1568009033481741)

**CD144**: Vascular endothelial (VE)–cadherin (CD144) is an adhesion molecule that mediates cell-cell contact between endothelial cells (https://doi.org/10.1182/blood-2005-05-1893)

**CD166**: CD166 is used as marker for human mesenchymal stromal cells contamination (such as fibroblasts) that are often co-isolated as an unwanted by-product from biopsy and they can rapidly overgrow in culture. (10.1016/j.gene.2020.100031)

**VIMENTIN**: Vimentin is recognized as a typical fibroblasts marker and is also common in endothelial cells. Borass et al., 2016 (https://doi.org/10.1038/srep30814) state: “The cytoskeletal filament vimentin is inherent to the endothelial phenotype and is critical for the proper function of endothelial cells in adult mice.”.
